# Rapid Clinical Management of Leishmaniasis in Emergency Department: A Case Report with Clinical Review of Recent Literature

**DOI:** 10.3390/biology9110351

**Published:** 2020-10-23

**Authors:** Andrea Piccioni, Federico Valletta, Christian Zanza, Yaroslava Longhitano, Enrico Torelli, Tommaso de Cunzo, Alessandra Esperide, Mattia Brigida, Veronica Ojetti, Marcello Covino, Samanta Taurone, Massimo Ralli, Marco Artico, Francesco Franceschi

**Affiliations:** 1 Department of Emergency Medicine, Fondazione Policlinico Gemelli-IRCCS, Università Cattolica del Sacro Cuore, 00168 Rome, Italy; andrea.piccioni@policlinicogemelli.it (A.P.); fede.valletta@gmail.com (F.V.); rikho88@hotmail.it (E.T.); tomdecunzo@gmail.com (T.d.C.); alessandraesperide@live.com (A.E.); mattia.brigida@gmail.com (M.B.); veronica.ojetti@gmail.com (V.O.); macovino@gmail.com (M.C.); francesco.franceschi@unicatt.it (F.F.); 2 Department of Emergency Medicine, Anesthesia and Critical Care, Michele and Pietro Ferrero Hospital, 12060 Verduno, Italy; 3 Department of Anesthesia and Critical Care Medicine, SS Antonio and Biagio and Cesare Arrigo Hospital, 15121 Alessandria, Italy; lon.yaro@gmail.com; 4 Department of Sensory Organs, Sapienza University of Rome, 00186 Rome, Italy; t.samanta@yahoo.it (S.T.); massimo.ralli@uniroma1.it (M.R.); marco.artico@uniroma1.it (M.A.)

**Keywords:** leishmaniasis, visceral leishmaniasis, leishmaniasis serology, signs of leishmaniasis, leishmaniasis differential diagnosis

## Abstract

**Simple Summary:**

In this article, we have briefly described the various forms of leishmania infection occur in emergency settings as well as the principal differential diagnoses, and we propose a decision algorithm to facilitate its early recognition in the emergency department (ED). Regarding the last point, the costs and validity of the most common modern diagnostic technologies have been examined, with particular attention to their sensibility and specificity; particularly, rk39-based RTD has been examined. To reinforce the importance of a quick diagnosis performed in the emergency room, we introduce a rather paradigmatic case report of a 19-year-old patient presenting with suspected lymphoproliferative disease and subsequently addressed to the incorrect hospital ward. As often happens, signs and symptoms tended toward the diagnosis of a hematologic disease rather than an infectious one: therefore, it is crucial to include a variety of diagnostic possibilities when a patient presents with fever and associated lympho-adenomegaly with minor symptoms: Leishmaniasis always needs to be considered among them.

**Abstract:**

Systemic or localized lympho-adenomegaly is a common cause of access to the emergency department (ED), and differential diagnosis is often complicated. The combination of anamnesis, physical examination, laboratory tests, and instrumental diagnosis are extremely important to orientate toward a rapid and correct therapy, even if a prompt discrimination of the etiology of this lymphadenomegaly is not often possible. Our aim with this review is to improve the management of a differential diagnosis between hematological and infective diseases as leishmaniasis in ED and suggest quick diagnostic techniques that might be useful for early identification. Together in the review, we describe a case report of a young man affected from visceral leishmaniasis who presented to our ED and was incorrectly addressed to the wrong ward for the study of his condition. Subsequently, we focus on the clinical presentation of visceral leishmaniasis and compare it to the most common differential diagnoses that are usually taken into account in the management of such patients.

## 1. Introduction

Systemic/localized lymphadenomegaly associated with fever and other nonspecific signs or symptoms are common causes of access to the ED, but the differential diagnosis is usually hard.

The combination of anamnesis, physical examination, laboratory tests, and instrumental diagnosis are extremely important to orientate toward a rapid and appropriate therapy, but currently, a prompt discrimination of the lymphadenomegaly etiology is not often possible.

The management of a differential diagnosis between hematological and infective diseases such as Leishmaniasis usually represents a challenge for the emergency physician; hence, we suggest a quick diagnostic test that might be useful for the early identification.

Leishmaniasis is a vector-born disease caused by a group of protozoan parasites belonging to the genus Leishmania [1].

Most Leishmania infections are zoonotic diseases except for those that have Leishmania *Tropica* and Leishmania *Donovani* as causative agents and are considered anthroponoses [2].

The infection begins with the bite of the vector, a female specimen of sandflies. In Africa, Europe, and Asia, the sand-fly Phlebotomus is widespread, while in the New Continent, the sand-fly Lutzomyia is responsible for the spread of Leishmania; however, they all are very similar morphologically.

After the parasite enters human cells, cutaneous macrophages phagocytize promastigote, which is the primary stage of the parasite. An immunocompetent system is commonly able to kill promastigotes, blocking the spread of parasites in other organs through cellular lysis. This phenomenon occurs in a small percentage of cases, where promastigotes resist the destruction and evolve into amastigotes, which replicate and provoke cellular lysis. The next progression step is the spreading of amastigotes into other reticular-endothelial system cells showing different clinical conditions as for gravity, clinical signs, and outcome [3].

*Cutaneous* Leishmaniasis is a severe but not deadly disease which usually manifests with self-limited ulcerative lesions that spontaneously heal in 6–18 months. Only 10% of cases evolve into systemic disease, which is potentially lethal (mucosal and mucocutaneous forms) [2,4].

The severity and chronicity of the skin lesion of leishmaniasis depend on two fundamental factors: the infecting species and the host’s immune response.

The lesion generally starts from the vector injection site and develops within about 2 weeks as papules or nodules; with the involvement of the lymph nodes draining the site of infection, eventually, a granuloma can develop from this lesion, and it will hesitate in healing, or it can ulcerate causing skin lesions that tend to become chronic [2].

*Visceral* Leishmaniasis is a severe form, typically occurring in rural areas, that requires prompt treatment to avoid fatal outcomes. The rate mortality is 10–20%, but this is just a poor estimation due to the lack of appropriate epidemiological methods and the numerous misdiagnosed cases [5,6].

The patient who approaches the emergency department with *visceral* leishmaniasis typically reports rapid weight loss in the preceding weeks. The presenting symptoms include fever, asthenia, weakness, anorexia, and night sweats. On physical examination, we may find hepatomegaly, splenomegaly, and lympho-adenomegaly.

A special class of patients with *visceral* leishmaniasis is represented by those with HIV: in these patients, visceral leishmaniasis infection is much more severe and leads to a progression of the acquired immunodeficiency, worsening the prognosis of HIV patients.

It has also been shown that HIV can lead to the re-activation of leishmania infection that was latent [7,8,9].

Leishmaniasis is characterized by an endemic diffusion in East Africa, Latin America, and South-East Asia, which are areas where malnutrition is associated with a high concentration of parasite; indeed, it is not well elucidated if the parasite is the etiology or a consequence of the poor nutritional status [10]. Despite the numerous efforts to contain the infection, developing countries failed to achieve their goal to eliminate Leishmaniasis as a public health issue by 2015 [11].

Nowadays, thanks to the increased migration of people, Leishmaniasis has recently spread worldwide, especially to several Mediterranean countries [1], forcing the consideration of Leishmaniasis as a potential differential diagnosis in patients presenting no specific symptoms associated to fever and lymphadenomegaly.

There is no effective pre-exposure prophylaxis or effective vaccine, and the only protective measures include individual devices (mask, gloves, distance) and public health measures.

In the case of leishmaniasis, people need to know the risk of infection and epidemiology:

(1) The infection is transmitted through sand-fly bites between dusk and dawn;

(2) Covering every part of body with clothing is protective, because sand-fly mouthparts do not penetrate clothing (in contrast, mosquito mouthparts do penetrate clothing). Moreover, clothes impregnated with permethrin make a stronger protection for exposed skin areas (face, neck, hands, forearms, feet, ankle, joints) and DEET (NN-diethyl-3-methylbenzamide) should be used an insect repellent.

Regarding public health prevention, the only measures are vector control (sand flies) and reservoir control (domestic and sylvatic animals) [12].

In areas where sylvatic rodents live and grow up, the reservoir control is not applicable due to the high concentration of rats to treat. Indeed, rats may harbor some of the Leishmania species, but it is not sure if they infect the sandflies. In contrast, the control of infection with domestic reservoir measures is more simple. In fact, the use of deltametrin-impregnated collars in dogs has been associated with decreased seroconversion rates of visceral leishmaniasis in humans and dogs, but its efficacy for the prevention of CL has not been evaluated [13].

At the moment, the diagnosis of a typical Leishmania infection is a true challenge for the physician, especially in the field of Emergency Medicine where time and precision run together; therefore, our review aims to analyze the latest literature in order to facilitate a prompt diagnosis of Leishmaniasis in the ED.

Another goal is to summarize the efficacy of some potential diagnostic tests that may provide a quick identification in the ED and distinguish *visceral* Leishmaniasis from other similar conditions. Before, we will introduce a case report of a particular form of Leishmaniasis.

## 2. Case Report

A 19-year-old man presented to our emergency department (ED) in January 2020 complaining of weight loss and asthenia for a few months. In anamnesis, there was a recent history of mononucleosis infection and the blood-test is shown above (Table 1). Physical examination revealed palpable spleen and protruding liver 4 cm below the costal arch, together with swollen latero-cervical and axillary lymph nodes bilaterally.

The patient underwent routine blood tests, chest X-ray, and abdominal ultrasound. Blood tests are reported as follows.

A chest x-ray showed no pathological changes, while an abdominal ultrasound detected an increased liver size (cranio-caudal diameter of about 18 cm), with regular margins and no focal lesions, and the spleen increased in size with biparietal diameter > 20 cm, free from focal lesions. The ultrasound also showed that the lymph nodes increased in size in the periportal area and at the level of the hepatic hilum and celiac tripod, with a maximum size of about 30 × 13 mm. Given the examinations and the clinical history, the patient was admitted to the Hematology Unit with suspected lymphoproliferative disease. A few days later, the diagnosis of *visceral* leishmaniasis was made based on the microscopic examination of bone marrow needle aspiration that described "intra and extracellular images consistent with Leishmania amastigotes". Treatment with Amphotericin B was started, and the patient was transferred to the Infectious Diseases department from which he was discharged after a week.

### 2.1. Case Discussion

From the retrospective analysis of the clinical case, it can be concluded that the signs, symptoms, laboratory, and ultrasound were all suggestive of Leishmaniasis. Nonetheless, since the clinical presentation was very suspicious for a lymphoproliferative disease, the young patient was first admitted to the Hematology department, which in the end did not prove to be the most appropriate department. Thus, it is mandatory to take into account Leishmaniasis whenever a patient presents with such symptoms and signs. However, since specific serological tests for visceral leishmaniasis cannot be determined in the ED so far, the physician should always consider it in the differential diagnosis and address the patient to a proper management.

### 2.2. Case Conclusion

**Figure 1:**
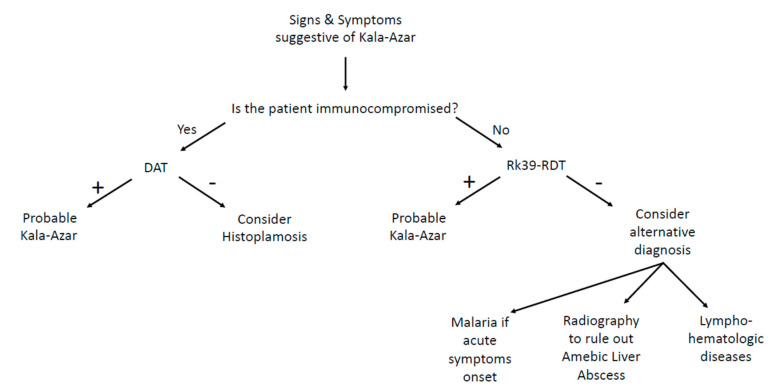
Clinical flowchart in emergency department.

Probably, we could improve the diagnostic–therapeutic procedure by performing targeted serological investigations such as antibody titers for visceral leishmaniasis, so as to reach the correct diagnosis more quickly and directly in the ED, with no delay in treatment. Starting a proper pharmacological therapy as soon as possible could prevent any of the life-threatening consequences that come along with the disease. Therefore, even though the prevalence of hematologic disease tends to push us in that direction, we must always consider the infectious etiology until proven otherwise (Figure 1).

## 3. Materials and Methods

Articles were identified using the electronic PubMed database through a comprehensive search conducted by combining key terms such as “leishmaniasis”, “visceral leishmaniasis”, “leishmaniasis serology”, “clinics of leishmaniasis”, and “leishmaniasis differential diagnosis”. Articles were screened for relevance. A full review was conducted for publications of the most relevant studies, including additional publications that were identified in the individual article reference lists.

Initially, the literature search was individually conducted by the single authors after a meeting to find out the work progression; all authors confronted each other to include only the most recent (published by the last 10 years) and most relevant articles. Finally, the bibliographies of all the selected articles were checked.

## 4. Results

### 4.1. Clinical and Diagnostic Tips for the Emergency Physicians

Leishmaniasis presents several clinical forms depending on the involved species, which are *Leishmania mexicana*, *Leishmania* (*Viannia*) *braziliensis*, *Leishmania panamensis*, *L. major*, and *L. tropica*. All of these cause the cutaneous Leishmaniasis, which is the consequence of an inefficient cellular-mediated response, and although it cannot be considered a life-threatening condition, people who are affected usually suffer from social stigmatization [3,14,15]. The incubation period lasts around 1–2 months, after which one or more reddish papulo-nodular lesions will appear on the inoculation point (face, neck, legs, or arms). These lesions can be ulcerated or not, and they can have different sizes.

Lympho-adenomegaly is frequently found near the skin lesion [15]: it is harmless, indolent, and self-limited within a few months; it is extremely rare that a disfiguring scar remains, although the skin is heavily infiltrated with parasites.

Approximately 10% of cutaneous forms evolve into mucocutaneous leishmaniasis (MCL), which is a disfiguring disease characterized by the progression into mucosal inflammation because of a combination of host cell-mediated immunity [15], parasite virulence, and inadequate treatment. For all of this, mucocutaneous leishmaniasis needs to be promptly treated [16]. Mucocutaneous leishmaniasis causes destructive lesions mainly on the lips, nasal septum, and palate. Lesions can easily be confused with other infectious diseases such as fungal infections. In most cases, the first symptom is nasal congestion, but with the disease’s progression, the symptoms worsen [2,15], and erythema, dysphagia, dysphonia, tooth loss, severe respiratory obstruction, and dyspnea may arise. When promptly recognized, MCL can be treated and solved before any consequence occurs [4].

*Visceral* leishmaniasis aka kala-azar occurs when a parasite spreads from the reticuloendothelial system to many organs. If left untreated, this is a harmful and potentially fatal condition that typically leads to death within 2 years. Early symptoms and signs include prolonged, persistent, and irregular fever, hepatomegaly, splenomegaly, pancytopenia, progressive anemia, and weight loss, despite not all of these features always being present at the same time [16].

As happened in our case report, nonspecific clinical presentation might often be misleading for the physician, who is primarily tempted to address the diagnosis toward a hematologic disease [15]. In fact, the most common signs—such as hepatomegaly, splenomegaly, and fever—are also present in infective, liver, autoimmune, and infiltrative diseases. Considering these signs intertwining, other pathologies must be considered in the diagnostic process.

### 4.2. Differential Diagnosis

The differential diagnosis of VL includes [17] the following:

***Malaria***—Both malaria and VL may present with fever, malaise, and splenomegaly; the main difference regards the symptoms onset: malaria generally occurs acutely, while VL tends to be chronic. The diagnosis of malaria is established by blood smear or rapid diagnostic testing.

***Histoplasmosis***—Patients with acute histoplasmosis present with fever, fatigue, hepatosplenomegaly, and pancytopenia; this is a disease that occurs in the setting of immunosuppression. The diagnosis is made by antigen testing, culture, or histopathology.

***Amebic Liver Abscess***—This pathology is characterized by one to two weeks of right upper quadrant pain and fever and sweating, malaise, weight loss, and anorexia. A rapid and cheap diagnostic toll is radiographic imaging.

***Schistosomiasis***—The principal manifestation is hepatosplenomegaly due to granulomatous inflammation and subsequent fibrosis of the periportal spaces of the liver, with subsequent portal hypertension. The diagnosis is established by the visualization of eggs on microscopy and/or serology.

***Lymphoma***—Lymphoma shares the principal symptoms of leishmaniasis as lymphadenopathy, hepatomegaly, splenomegaly, cytopenia, fever, night sweats, and weight loss. The only valid diagnosis is established by histopathology.

***Tuberculosis***—Only symptoms of extrapulmonary tuberculosis may present with seeding of nearly any organ of the body, including hepatic and/or splenic disease. The diagnosis is established by culture of acid-fast bacilli from the sputum or other fluid/tissue.

In lympho-hematologic diseases, there is a high hepatomegaly/splenomegaly prevalence that drives the physicians to consider more such a disease than an infectious one.

The onset of visceral Leishmaniasis can be acute and hard to make; anyway, no delay is allowed for treatment to prevent a potentially fatal evolution [18] (Table 2).

### 4.3. How Physicians Diagnose Visceral Leishmaniasis in ED

First, nonspecific symptoms are more common than specific ones, and the suspicion of visceral Leishmaniasis must be confirmed through accurate diagnostic tests.

The traditional diagnostic method is the direct amastigote microscope visualization of biopsied samples of spleen, lymph nodes, bone marrow, or liver. However, sensitivity strictly depends on the analyzed tissue, ranging from 50% to 90%; even blood samples have a low sensitivity, except in HIV-positive patients who have a higher parasitemia level [19,20]. Polymerase Chain Reaction (PCR) on bone marrow, peripheral blood, or buffy coat samples has a sensitivity > 95% both for *L. donovani* in Asia and east Africa and *L. Infantum* in the Mediterraneum, but the low specificity, the high costs, and the complexity of the technique make it hard to be introduced in the diagnostic algorithm [21]. A latex antigenic test that aims to detect a heat-stable low molecular weight carbohydrate antigen in urine is now available, but it is rarely used in clinical practice due to a low sensibility (64%), despite an excellent specificity (93%) [22].

Several serologic tests are available, including the enzyme-linked immunosorbent assay (ELISA), the indirect fluorescent antibody test (IFAT), the indirect hemagglutination assay (IHA), and Western blot (WB). All of these methods involve antibody detection tests, hence sharing the same issues. Indeed, both sensitivity and specificity range from 80% to 100%, techniques are expensive and complicated to perform (limiting their use in endemic countries), asymptomatic infected patients often result positive, and sensitivity is much lower in immunocompromised patients [23]. An RK39-based rapid diagnostic test (RDT) is currently widely used in North America. RK39 is a 39-amino-acid protein produced by a Brazilian *L. infantum*/*chagasi* strain. The test has shown a sensitivity of 97% in the Indian subcontinent, although it resulted only 85% sensitivity in eastern Africa. More recently, an RK28-RDT has been introduced, and it has maintained a high sensitivity in India while improving its sensitivity to 95% in eastern Africa [24,25,26]. However, RTDs have the same limitations of other serologic tests, except for them being cheaper and easier to use [26].

Antigen-based immune-chromatic tests consist of analyzing a peripheral blood drop sample with a nitrocellulose membrane pre-coated with the RK39 antigen. The test is quick and easy to perform, and it has been largely used worldwide, especially in rural areas with a lack of health facilities. However, sensitivity strongly varies based on the tested area, with values ranging from 92.8% to 100% in India to 36.8% to 92% in Brazil and East Africa; therefore, a negative result cannot rule out the diagnosis of visceral leishmaniasis, especially in patients with HIV. In addition, specificity is limited by the cross-reaction with a large number of different diseases, such as infective endocarditis, hepatic insufficiency, malaria, enteric fever, disseminated tuberculosis, lymphoma, sepsis, and toxoplasmosis [27].

The diagnosis of visceral leishmaniasis is particularly challenging in immunocompromised patients for whom serologic tests are usually unable to detect the disease due to the low antibodies levels [23,24,25,26,28]. The best results have been obtained with WB, which showed a sensitivity between 75% and 91%. However, due to the lack of data comparing WB and direct agglutination test (DAT), it is not possible to recommend the use of WB for immunocompromised patients: in these individuals, it would be preferred to perform two serologic tests and PCR [29].

The direct agglutination test (DAT) is widely used in South America, Iran, and in some European countries [30,31,32,33]. This is a semi-quantitative test that aims to detect if agglutination occurs when serial dilutions of patient’s serum are mixed with stained killed *Leishmania* sp. promastigote. Therefore, DAT is not influenced by the involved species (*L. donovani* or *L. infantum*) [34]. Sensitivity is 70.5–99% and specificity is 82.2–100%, with high values even for HIV-positive individuals (89.1–91.3% and 89.3–89.7%, respectively), thus making it a very useful test for the diagnosis of visceral leishmaniasis in a large population with or without HIV [35,36,37,38]. However, false positive can result in asymptomatic patients or individuals affected by malaria or other parasites [23,31,33,39]. In 2006, Chappuis and coworkers conducted a meta-analysis to compare the diagnostic performances of the DAT test and the rK39 dipstick. They only analyzed studies conducted on patients with a certain diagnose of VL by splenic aspirate and finally included 30 studies evaluating the DAT test and 13 evaluating the RK39 test. The results showed that both tests perform good or excellent for the diagnosis of VL, with a sensitivity of 94.8% and a specificity of 85.9% for DAT, and a sensitivity of 93.9% and a specificity of 90.6% for rk39 dipstick (Figure 1) [40].

### 4.4. Pharmacological Basis

As previously mentioned, visceral Leishmaniasis is a life-threatening condition marked by a mortality rate above 60–90% without treatment [41]. Once identified and before any control regarding the infection, the first purpose of the physician in the emergency department should aim at setting up a proper supporting therapy to correct the nutritional status, anemia, and hemorrhagic complications, the latter being particularly lethal for the patients [42].

The specific treatment lasts a long time and requires the use of infusion drugs that are expensive and usually toxic. Amphotericin B is the most widely used drug in Europe and North America; however, the high costs limit its use in developing countries [43,44].

Pentavalent antimonials are still the most common drugs in the world for VL treatment, even if no longer in monotherapy but in combination with miltefosine and paromomycin [45]. Combined treatment limits the development of resistance, has a shorter duration, improves compliance, and reduces costs [43]. However, combination therapy guidelines do not yet exist [45].

### 4.5. Assessing Response to Treatment

After pharmacological treatment, the resolution occurs step by step starting from fever fading typically within one or two weeks, decrease in spleen size within a month, and weight gain within 3 months. Patients do not need parasitological confirmation, but they should be followed for at least 12 months and instructed to return if symptoms recur [17].

The follow-up management is different for immunocompetent and immunocompromised patients. For the first ones, most relapses occur within 6 to 12 months of completion of treatment (but in one observational study with extended follow-up, relapse symptoms were seen up to 18 months post-treatment). For immunocompromised patients, the follow-up should last for a minimum of one year, ideally lifelong or until effective immune reconstitution, to assess for symptoms of post-treatment relapse [46].

In patients with equivocal clinical response (e.g., no decrease in spleen size, continued fever) or with a suspected relapse, bone marrow or splenic aspirate should be performed to confirm VL or to evaluate for alternative diagnoses or to evaluate the coexistence of two pathologies (one cause and another one consequence).

For the test of cure, conventional serologic tests are not useful because they remain positive until one year after treatment [47,48]. The urine KAtex assay detects parasite antigen in urine and becomes negative more rapidly than serologic assays but has poor sensitivity for diagnosis and is not widely available [49].

Currently, for the test of cure, Anti-rK39 immunoglobulin (Ig)G1 is the best choice. In fact, in a small study of 37 patients with demonstrated clinical response to treatment, 81% of those without relapse symptoms were negative to anti-rK39 IgG1 ELISA at 6 months, and 85% of those with clinical signs of relapse had a positive result (Figure 1) [50].

Adjusting therapy, some immunocompetent patients do not respond to initial therapy with liposomal amphotericin; actually, there are no clinical trials to determine the optimal regimen. In these cases, the physician can choose a higher dose or longer course of liposomal amphotericin or a combination regimen with miltefosine or pentavalent antimonial compound [17].

In immunocompetent patients, if the first-line therapy was miltefosine or a pentavalent antimonial compound and the patients do not respond to initial therapy, a valid alternative regimen could be liposomal amphotericin.

In the case of immunocompetent patients with VL who respond to initial therapy but subsequently relapse, treatment should include an alternative drug or a longer course of therapy with the initial drug. If liposomal amphotericin was the drug used for initial therapy, the use of a higher total dose (e.g., 30 to 40 mg/kg) may be warranted.

Immunocompromised patients with VL who do not respond to initial therapy can be managed by retreatment with liposomal amphotericin at the same or a higher total dose or with a combination such as liposomal amphotericin B plus miltefosine [51].

## 5. Discussion

This review aims to help the physician make a prompt identification of a case of visceral leishmaniasis in the emergency department, so as to avoid some potentially fatal complications associated with the disease, as well as unnecessary investigations arising from a wrong diagnosis. 

The problem has long concerned only some endemic areas, such as East Africa, Latin America, and South-East Asia. However, due to the migration flows of the last decades, leishmaniasis has recently spread worldwide, especially to several Mediterranean countries. This makes it necessary to include it when performing a differential diagnosis for a patient with a compatible clinical presentation in the emergency room.

Early symptoms and signs of leishmaniasis include fever, hepatomegaly, splenomegaly, pancytopenia, progressive anemia, and weight loss. Such a clinical presentation can easily be misleading, and the physician is usually oriented toward a diagnosis of a hematologic disease [52].

To date, there are no specific tools for a secure diagnosis of leishmaniasis in the ED. The current gold standard is polymerase chain reaction (PCR) performed on bone marrow, peripheral blood, or buffy coat samples. This technique has shown a good sensitivity but a low specificity. In addition, it is a complicated procedure that takes a long time to be performed.

Another valuable diagnostic option points to serologic tests, such as the enzyme-linked immunosorbent assay (ELISA), the indirect fluorescent antibody test (IFAT), the indirect hemagglutination assay (IHA), and Western blot (WB). All of these methods involve antibody detection techniques and share the same problems that limit their use: indeed, they are expensive and complicated to perform in urgency. In addition, their sensitivity dramatically falls in immunocompromised patients.

A new diagnostic method for leishmaniasis is represented by the rk39-based rapid diagnostic test (RDT), which is currently widely used in North America. Despite sharing the same limitations of other serologic tests, RDT is cheaper and easier to perform in the laboratory, and it has shown high sensitivity and specificity, making it a valuable option for the ED setting [53].

Visceral leishmaniasis is a potentially fatal disease, with 75 to 95% of untreated cases leading to death usually because of bacterial superinfection. If promptly and correctly treated, mortality falls to 5%.

Guidelines for a proper therapy do not yet exist [54]. However, some fundamental measures must be taken during the initial evaluation of a patient with a suspicion of leishamaniasis, such as supporting the nutritional status and correcting anemia and preventing hemorrhagic complications [42].

Thus, it is evident how the introduction of a diagnostic tool that might offer a fast diagnosis of visceral leishmaniasis in the ER would dramatically improve the management of the disease and reduce the associated morbidity and mortality.

A prompt identification would also reduce the unnecessary costs arising from an improper admission of the patient to the wrong department.

Our proposal leans toward the improvement of the diagnostic–therapeutic procedure by performing alternative targeted serological investigations so as to reach the correct diagnosis more quickly in the ER and send the patient to the most appropriate department for further evaluation.

In particular, because of its accuracy and speed of execution, we suggest the introduction of rk39-based RTD for individuals who present with signs and symptoms compatible with visceral leishmaniasis in the ED.

## 6. Conclusions

Considering the available tools in the ED, our first concern should always be to reduce the risk of a rapid worsening of the clinical setting. For this purpose, the correct diagnosis of the underlying condition turns out to be mandatory in order to address the patients to the right department.

As seen in our case report, VL presents with a very nonspecific cohort of signs and symptoms, which can be addressed to a wide range of different and more common conditions. Therefore, it becomes mandatory for the ED physician to include VL in the differential diagnosis. However, even in front of a very suspicious case for VL, the ED physician does not have enough instruments for a quick and certain diagnosis.

The current gold standard for VL diagnosis is the microscopic identification of the amastigote in splenic, lymph nodes, bone marrow, or liver samples. It is evident that such a procedure is time-dependent, very complex, and cannot be performed in the ED. 

Nevertheless, recent studies show that some rapid test such DAT or RK39 dipstick have high sensibility and good specificity for VL (Figure 1); moreover, they are cheap and easy to perform and might be optimal for an initial screening in the ED when there is a high VL clinical suspicion. More studies investigating the diagnostic accuracy of these rapid tests are needed in order to validate their applicability in the ED at our latitudes. Hence, we suggest the introduction of DAT or rK39 in the ED setting for the initial screening of VL, when the clinical pattern is suggestive.

Other than kit to detect Leishmania, currently, the research is developing a vaccine for different human VL. The principal targets are as follows: (1) reduce the infectiousness of infected individuals toward sand flies, (2) reduce the risk of developing symptoms after infection, (3) reduce the risk of developing post-kala-azar dermal leishmaniasis (PKDL), and (4) develop transient immunity.

Even though vaccines are not yet available for implementation, their development should be pursued, as their potential impact on transmission can be substantial both in decreasing incidence at the population level as well as in sustaining the Indian Sub Continent elimination target when other interventions are halted [55].

In the diagnosis of leishmania, laboratory results are very important, but in an emergency setting, the clinical part with differential diagnoses remain the only weapons to quickly defeat the disease. 

## Figures and Tables

**Table 1:** Blood tests of case report.

Laboratory Tests	
hemoglobin 9.1 g/dL– MCV 75.4 fL	white blood cells 2650/mm^3^ (N 23%, L 48%, M 21%)
LDH 327 IU/L	alkaline phosphatase 162 IU/L
AST 52 IU/L	ALT 59 IU/L
c reactive protein 43.1 mg/L	d-dimer 4257 ng/mL

**Table 2:** Summary of the most common differential diagnosis that could mimic leishmaniasis.

	Splenomegaly	Hepatomegaly	Fever	Weight Loss	Cytopenia
Liver diseases (e.g., fibrosis, cirrhosis)	Common	Common	Rare	Common	Common
Hematologic Malignancies					
Chronic leukemia	Common	Common	Rare	Common	Common
Lymphoma	Common	Common	Common	Common	Common
Myeloproliferative diseases	Common	Common	Rare	Common	Common
Multiple myeloma	Common	Possible	Common	Common	Common
Autoimmune cytopenia (e.g., ITP^1^, AIHA^2^)	Common	Rare	Possible	Rare	Common
Infectious diseases					
Viral (e.g., hepatitis, EBV, HIV/AIDS)	Common	Common	Common	Common	Common
Bacterial (e.g., mycobacteria, leptospirosis, brucellosis)	Possible	Possible	Common	Common	Common
Parasitic (e.g., malaria, schistosomiasis)	Common	Possible	Common	Rare	Rare
Fungal (e.g., histoplasmosis)	Common	Possible	Common	Common	Common
Infiltrative diseases (i.e., amyloidosis, sarcoidosis, Felty sindrome, SLE^3^, HLH^4^)	Common	Possible	Possible	Possible	Common
